# Regulation of base excision repair during adipogenesis and osteogenesis of bone marrow-derived mesenchymal stem cells

**DOI:** 10.1038/s41598-023-43737-z

**Published:** 2023-09-29

**Authors:** Min Kim, Hyun-Jin Jang, Song-Yi Baek, Kyung-Jin Choi, Dong-Hee Han, Jung-Suk Sung

**Affiliations:** Department of Life Science, Dongguk University-Seoul, Biomedi Campus, 32 Dongguk-ro, Ilsandong-gu, Goyang, Gyeonggi-do 10326 Republic of Korea

**Keywords:** Genetics, Stem cells

## Abstract

Bone marrow-derived human mesenchymal stem cells (hMSCs) can differentiate into various lineages, such as chondrocytes, adipocytes, osteoblasts, and neuronal lineages. It has been shown that the high-efficiency DNA-repair capacity of hMSCs is decreased during their differentiation. However, the underlying its mechanism during adipogenesis and osteogenesis is unknown. Herein, we investigated how alkyl-damage repair is modulated during adipogenic and osteogenic differentiation, especially focusing on the base excision repair (BER) pathway. Response to an alkylation agent was assessed via quantification of the double-strand break (DSB) foci and activities of BER-related enzymes during differentiation in hMSCs. Adipocytes showed high resistance against methyl methanesulfonate (MMS)-induced alkyl damage, whereas osteoblasts were more sensitive than hMSCs. During the differentiation, activities, and protein levels of uracil-DNA glycosylase were found to be regulated. In addition, ligation-related proteins, such as X-ray repair cross-complementing protein 1 (XRCC1) and DNA polymerase β, were upregulated in adipocytes, whereas their levels and recruitment declined during osteogenesis. These modulations of BER enzyme activity during differentiation influenced DNA repair efficiency and the accumulation of DSBs as repair intermediates in the nucleus. Taken together, we suggest that BER enzymatic activity is regulated in adipogenic and osteogenic differentiation and these alterations in the BER pathway led to different responses to alkyl damage from those in hMSCs.

## Introduction

Human bone marrow-derived mesenchymal stem cells (hBMMSCs), one of the progenitor cells in adult mammals, are usually quiescent in the bone marrow and differentiate into various lineages, namely osteogenesis, adipogenesis, chondrogenesis, myogenesis, and neurogenesis^1–4^. Especially, hMSCs selectively differentiate into adipocytes or osteoblasts in a reciprocal pathway^5^. Adipocytes negatively influence osteogenesis by the production of fatty acids through lipolysis. It has been reported that bone density is reduced during aging in humans due to decreased osteoblasts and an increased number of adipocytes with age^6^. Moreover, the adipocytes in bone marrow secrete various adipokines to regulate the bone microenvironment, including stimulating and suppressing osteoclastogenic and hematopoietic signals, respectively^7^. Osteoblasts contribute to bone formation by synthesizing collagen and mineralizing the bone matrix^8^. Overall, the microenvironment within the bone maintains the balance among the multiple cell types of the niche, including stromal cells, adipocytes, osteoblasts, and hematopoietic cells. These cell types interact with hBMMSCs to maintain skeletal integrity via continuous skeleton remodeling, which is essential for the long-term maintenance of bone throughout life^9^.

Alkylating agents, such as methyl methanesulfonate (MMS), constitute a significant proportion of chemotherapeutics against cancer cells^10,11^. However, these agents also considerably threaten healthy cells, including bone marrow and intestinal epithelial cells^12^. Chemotherapeutic alkylating agents such as MMS can impair homeostasis and intracellular balance^13,14^. In the bone, chemotherapeutic drugs inhibit bone mineralization by damaging bone cells, such as osteoblasts, which contribute to bone formation and remodeling through the synthesis of bone extracellular matrix proteins^15,16^. These side effects of alkylating agents result in bone-related disorders, such as osteoporosis, which is characterized by reduced bone mineral density and increased fracture risk^17,18^. Alkylating agents transfer alkyl groups onto DNA bases, thereby altering DNA structure and interrupting the cellular function of the altered gene^12^. DNA repair maintains genomic stability and loss of DNA repair capacity results in genetic instability that can lead to a deterioration of cellular function^19^. Among the DNA repair systems, the base excision repair (BER) pathway corrects alkylated bases, oxidative lesions, and basic sites in nuclear and mitochondrial DNA^20,21^. Recent studies have suggested that the high efficiency of DNA damage repairing in hMSCs is reduced during their differentiation^22^. In addition, it has been reported that the BER pathway enzymes 8-oxoguanine glycosylase (OGG1) and Nei-like DNA glycosylase 1 (NEIL1) are regulated during neurogenesis^23,24^, and X-ray repair cross-complementing protein 1 (XRCC1) and ligase 1 are downregulated during myogenesis^25–27^.

The BER process accompanies the sequential actions of multiple enzymes, generally starting with the recognition and removal of a damaged base by DNA glycosylases^28,29^. Monofunctional glycosylases, including *N*-methylpurine-DNA glycosylase (APNG) and uracil-DNA glycosylase (UDG), generate abasic sites, and then AP endonuclease (APE1) incise the abasic sites and produce 3′-hydroxyl termini^30^. Bifunctional glycosylases, such as OGG1 and NEIL1, have glycosylase activity and also possess AP lyase activity, which can cleave the phosphodiester bonds in abasic sites to create a single-strand break without the need for an APE1^31^. These strand breaks, one of the repair intermediates, cause genotoxicity when accumulated but are usually eliminated during the gap-filling step^32^. At this step, poly(ADP-ribose) polymerase-1 (PARP1), X-ray cross-complementing protein-1 (XRCC1), and DNA ligase IIIα (LIG3) participate. XRCC1 is a scaffold protein that stabilizes the polβ/XRCC1/LIG3 complex during the insertion of the correct nucleotides into the gaps in DNA and enhances the handoff of nicked-repair products to the final ligation step^33^. The maintenance of genome integrity is a crucial task for stem cells because genetic modification could negatively influence the cell, such as during aging or tumorigenesis^34,35^.

In this study, we focused on how the MMS-induced DNA damage repair processes are regulated in adipogenic and osteogenic differentiation of hMSCs. This study demonstrates the correlation between DNA repair capacity and the sensitivity of differentiated cells to DNA damage. Here, we aimed to investigate whether that BER enzymatic activity is regulated differently by adipogenesis and osteogenesis. Besides, we hypothesized this alternative regulation of BER pathway could result in distinct responses to alkyl DNA damage in two types of differentiated cells derived from the same hMSCs. The results contribute to the understanding of the alteration in DNA-damage repair proteins during osteogenic and adipogenic differentiation of hMSC. Our findings may help to identify the cause of chemotherapy-induced bone damage. Additionally, this study provides key concepts for the proper use of alkylating chemotherapeutics.

## Results

### Adipogenic and osteogenic differentiation of hMSCs

**Figure 1:**
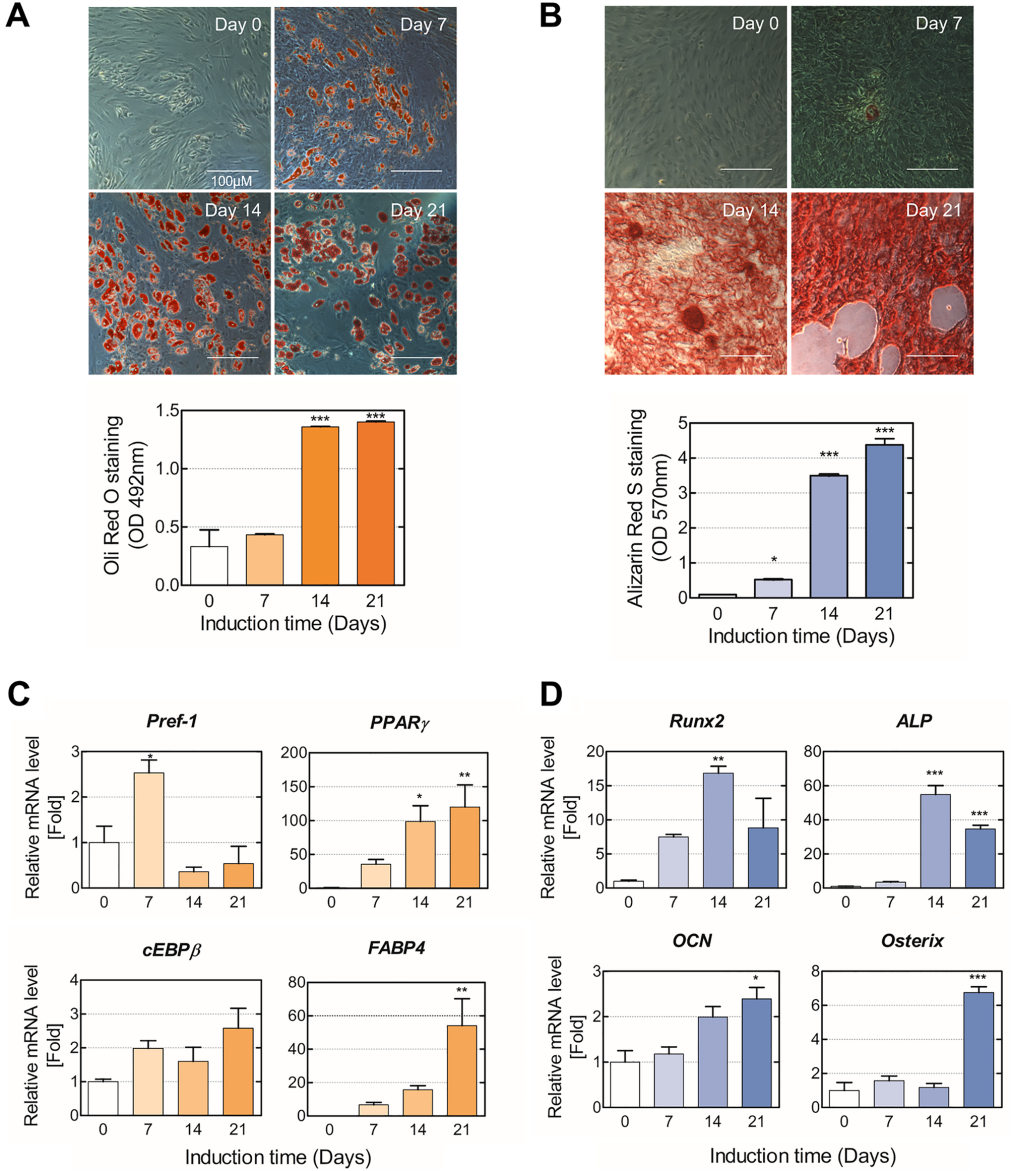
Adipogenic and osteogenic differentiation of hMSCs. (**A**) Adipogenic differentiation and (**B**) osteogenic differentiation were evaluated via ORO and ARS staining on days 0, 7, 14, and 21 post-inductions. Images were taken using a phase-contrast microscope, and the stained dye was dissolved and then quantified into relative values compared with the control. (**C**) The relative expression levels of Pref-1, C/EBPβ, PPARγ, and FABP4 during the adipogenic differentiation of hMSCs were evaluated via RT-qPCR. (**D**) The relative expression levels of Runx2, ALP, OCN, and osterix during the osteogenic differentiation of hMSCs were evaluated via RT-qPCR. RT-qPCR reading for each target was normalized to the expression level of GAPDH. Values are means ± SEM (n = 3). **p < 0.01, compared with the MSC group.

In this research, DNA damage response against an alkylating agent was investigated during adipogenic and osteogenic differentiation of hMSC. Therefore, it was important to validate the stage of differentiation. First, phenotypical changes and lipid accumulation during adipogenesis were examined by ORO staining, which stains the cellular lipid droplets as an indicator of adipogenic differentiation (Fig. 1). The lipids droplets were increased in size and number upon the treatment of the cells with AIM until day 21. The absorbance of stained ORO was gradually increased during the period of adipogenic differentiation and markedly enhanced after day 14 following adipogenic induction. Bone mineralization and calcification are developed during the osteogenic differentiation, and Ca^2+^ accumulates in the extracellular matrix of osteoblast^36^. To evaluate bone mineralization during the osteogenic differentiation of hMSC, calcium deposition was measured by ARS, which stains calcium^37^. The results show that ARS stained highly by day 21 but barely stained by day 7 of osteogenic differentiation.

To validate the molecular characteristics of the cells in each stage, the mRNA level of adipogenic and osteogenic markers were analyzed. The level of mRNA for preadipocyte factor-1 (Pref-1), which is known to be robustly upregulated in pre-adipocytes but inhibited following maturation into adipocytes, was highly expressed at day 7, whereas it was largely decreased after 14 days of adipogenic differentiation. Furthermore, mature adipocyte-specific markers were evaluated to determine the terminally differentiated phase of adipocytes; the key transcription factor of adipogenesis, CCAAT/enhancer-binding protein-beta (C/EBPβ) and peroxisome proliferator-activated receptor gamma (PPARγ), and fatty-acid binding protein (FABP4). As a result, the mRNA levels of PPARγ and FABP4 were significantly increased during the differentiation period, especially on day 21. In the case of osteogenesis, runt-related transcription factor 2 (Runx2) and alkaline phosphatase (ALP) are induced during the early stage of osteogenic differentiation, and their expression levels are decreased in mature osteoblasts. Osteocalcin (OCN), secreted by mature osteoblasts, is a bone-derived hormone involved in bone remodeling^38,39^ and is considered a mature osteoblast marker with osterix. The results show that the expression of Runx2 and ALP was significantly induced by day 14, and OCN and osterix were maximally expressed on day 21. Taken together, the cells treated for 7- and 21-days following induction of adipogenic differentiation were identified as pre-adipocytes and mature adipocytes, respectively. In addition, cells treated for 14 and 21 days after induction of osteogenic differentiation were identified as pre-osteoblasts and mature osteoblasts, respectively.

### Tolerance of hMSCs, adipocytes, and osteoblasts to alkylating agents

**Figure 2:**
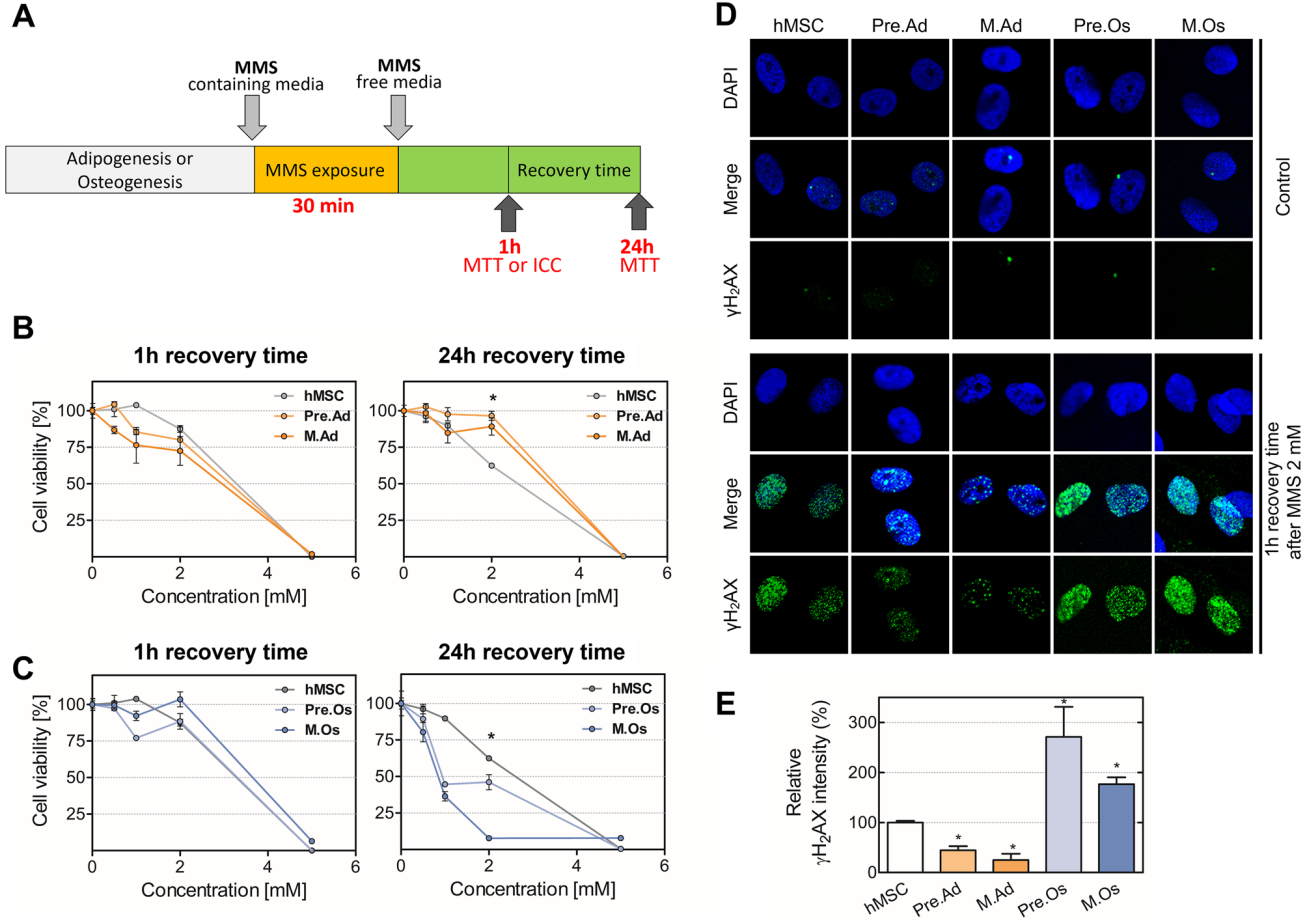
Cell viability and DNA damage of hMSCs and its differentiated cells upon exposure to an alkylating agent. Cytotoxicity of the alkylating agent was evaluated during adipogenic and osteogenic differentiation of hMSCs. (**A**) The scheme of MMS treatment was shown. The viability of hMSCs, (**B**) pre-adipocytes, mature adipocytes, (**C**) pre-osteoblasts, and mature osteoblasts was measured after 0.5, 1, 2, 5 mM of methyl methanesulfonate (MMS) exposure. After treatment with the indicated concentration of MMS for 30 min, viable cells were quantitated via the MTT assay after 1 h and 24 h of recovery time. (**D**) Immunocytochemical analysis of hMSCs and differentiated cells for γH2AX. Representative cells were treated with 0 mM (Control) or 2 mM MMS for 30 min and incubated in MMS-free media for 1 h, as observed under a confocal microscope. Cells were pre-permeabilized, detergent-washed, fixed, and then immune-stained using an anti-γH2AX antibody (green), and counter-stained with DAPI (blue). (**E**) The quantified value of each fluorescence is shown in the graph. *p < 0.05, compared with the MSC group. hMSC, proliferating hMSC; Pre.Ad, pre-adipocytes; M.Ad, mature adipocytes; Pre.O, pre-osteoblast; M.Os, mature osteoblast.

hMSCs have been shown to have a remarkable ability for DNA repair and self-renewal to regulate tissue homeostasis and regeneration^40^. In addition, hMSCs are well known for their resistance to exposure to chemotherapeutics, such as cisplatin, γ-irradiation, and etoposide. Therefore, to investigate the changes of sensitivity to alkylating agents according to modulation of the BER pathway during differentiation in hMSCs, cells differentiated into the indicated phase were analyzed after exposure to MMS for 30 min followed by 1 h or 24 h of recovery (Fig. 2A–C). The cell viability at each differentiation phase was decreased by the MMS treatment dose-dependently. Especially after 1 h recovery time following 2 mM MMS treatment, more than 75% of cells survived in all cell types, but adipocytes and osteoblasts differentiated from hMSCs responded differently to the MMS-induced damage after 24 h recovery time. In adipocytes, approximately 70% survived 1 h after exposure to 2 mM MMS, and after 24 h, no more cells died. However, in osteoblasts, 80% were alive 1 h after exposure to 2 mM MMS, but after 24 h, most fully differentiated cells were dead. Interestingly, these cytotoxicity results show that proliferating hMSCs are more vulnerable to MMS-induced cellular damage than differentiated adipocytes. Moreover, osteogenic differentiated cells were significantly more sensitive to MMS than hMSC. This means that the cells respond differently according to their differentiated type, even though they are all derived from the same cells.

We next evaluated the DNA double-strand breaks (DSBs) after MMS exposure according to the adipogenic differentiation status of hMSCs (Fig. 2D and E). DNA damage triggers an orchestrated assembly of proteins that are related to DNA damage sensors, signal transducers, repair effectors, cycle arrest, or apoptosis effectors^41^. Especially, DSBs that threaten genome homeostasis are powerful inducers of DNA damage response (DDR). To analyze the DNA damage, phosphorylation of histone H2AX (γH2AX) was evaluated through immunocytochemistry. H2AX is a member of the histone H2A family and is phosphorylated to induce DNA repair protein recruitment, initiating DDR upon DNA damage. 2 mM MMS significantly induced an accumulation of γH2AX foci with high intensity in hMSCs. Accumulation of γH2AX foci in adipocytes was significantly decreased approximately six-fold compared with hMSCs. On the contrary to adipocytes, osteoblasts with MMS treatment have approximately three-fold increased numbers of γH2AX foci compared with hMSCs.

Taken together, these results indicate that osteoblasts are most sensitive to MMS, and undifferentiated hMSCs are more sensitive to the alkylating agent MMS than differentiated adipogenic cells, as suggested by the accumulation of DNA strand breaks.

### Regulation of DNA monofunctional glycosylases in the BER pathway during adipogenesis and osteogenesis

**Figure 3:**
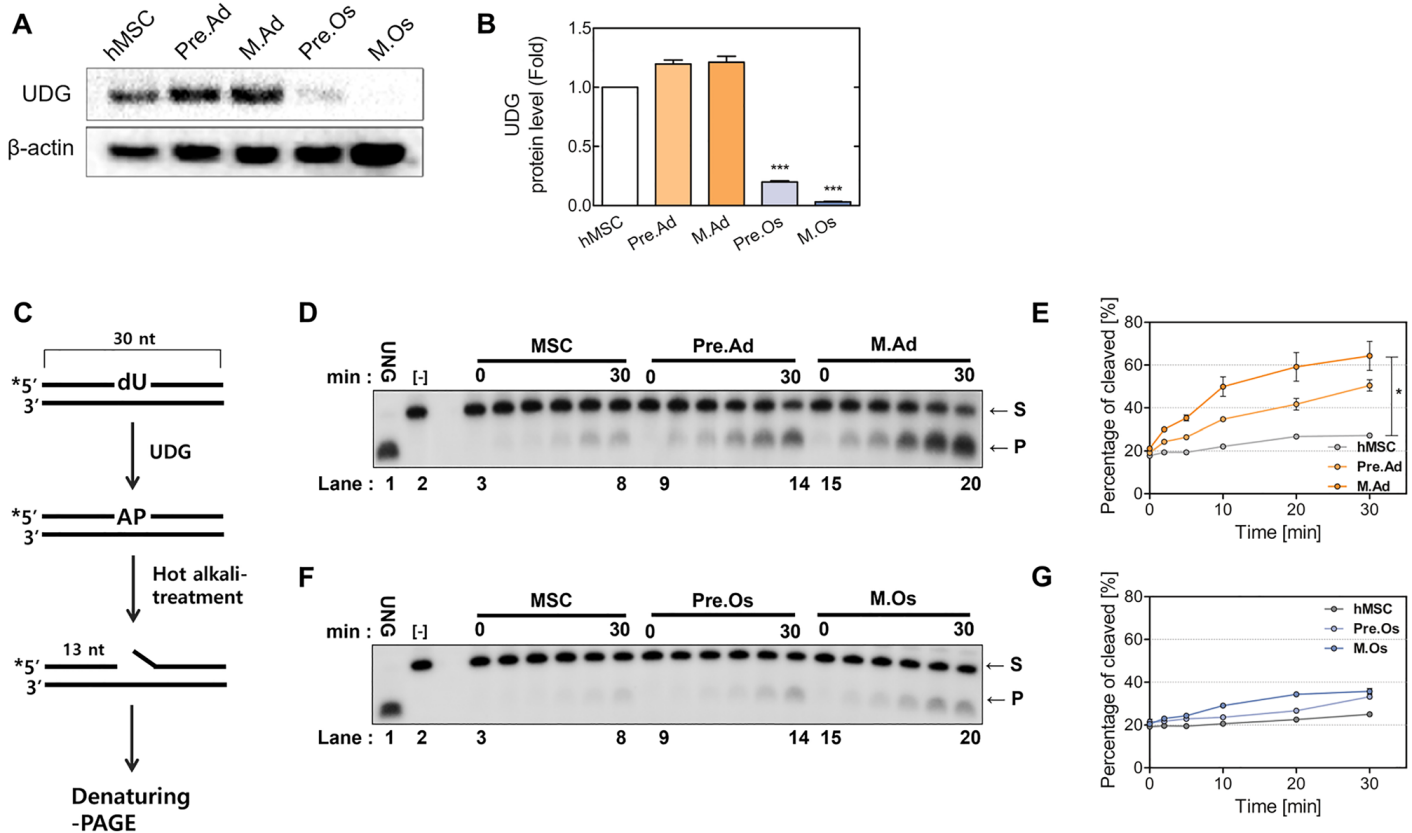
UDG expression and activity during the adipogenesis and osteogenesis of hMSCs. (**A**) Western blot showing the protein level of UDG, and (**B**) quantitation results. Full-length blots/gels are presented in Supplementary Fig. 3. (**C**) Schematic representation of the UDG-activity assay. The 30-bp duplex DNA substrates containing single deoxy-uracil residue were labeled with [γ-32P]ATP at the 5′-end. The AP site generated through the enzymatic removal of the uracil residue was hydrolyzed to produce the 13-bp cleaved products. Enzyme reactions contained 50 ng whole-cell extracts of (**D**) the adipogenic and (**F**) osteogenic differentiated cells at each phase in the reaction buffer. Purified *E. coli* UDG (UNG) was used for generating 13-mer products as a positive control. The products were resolved using denaturing 15% PAGE and detected using autoradiography. (**E** and **G**) Each activity was quantified and plotted into a graph. MSC, proliferating hMSC; Pre.Ad, pre-adipocytes; M.Ad, mature adipocytes; Pre.O, pre-osteoblast; M.Os, mature osteoblast; S, substrate; P, product.

To investigate whether the variation in the levels of the major proteins involved in the BER pathway during adipogenic differentiation affects the accumulation of DSBs, the expression and activity of each enzyme involved in DNA repair are analyzed. First, DNA glycosylase, UDG, and OGG1, an enzyme that selectively cleaves DNA substrates, can initiate the next step of the BER pathway by generating AP sites^42^. UDG was increased during adipogenic differentiation and decreased during osteogenic differentiation. Especially, UDG was upregulated 1.3-fold in mature adipocytes but significantly downregulated in osteoblasts, compared with the level in hMSCs (Fig. 3A and B). To study this enzymatic reaction, we ^32^P-labeled a specific DNA substrate containing the uracil residue and conducted an enzymatic cleavage assay by using whole-cell extracts from each phase of differentiation. To determine the initial rates of UDG activity, time-course enzymatic reactions were conducted for various incubation periods with the cell-free extracts from each phase of differentiation. The enzymatic activity showed a significantly different removal ability for a uracil residue from a U/G mispair, according to each phase. The UDG activities were prominently increased time-dependently during the adipogenic differentiation, whereas no significant change was observed during osteogenesis. Kinetic studies indicated remarkable stimulation of UDG activity during the developmental phase of adipogenesis. These results show that the protein expression of UDG, which removes alkyl-DNA lesions, was upregulated through adipogenic differentiation, and the UDG of adipocytes efficiently catalyzes the incision of uracil residues from mispaired DNA compared with proliferating hMSCs.

### Regulation of the DNA-ligation activity during adipogenesis and osteogenesis

**Figure 4:**
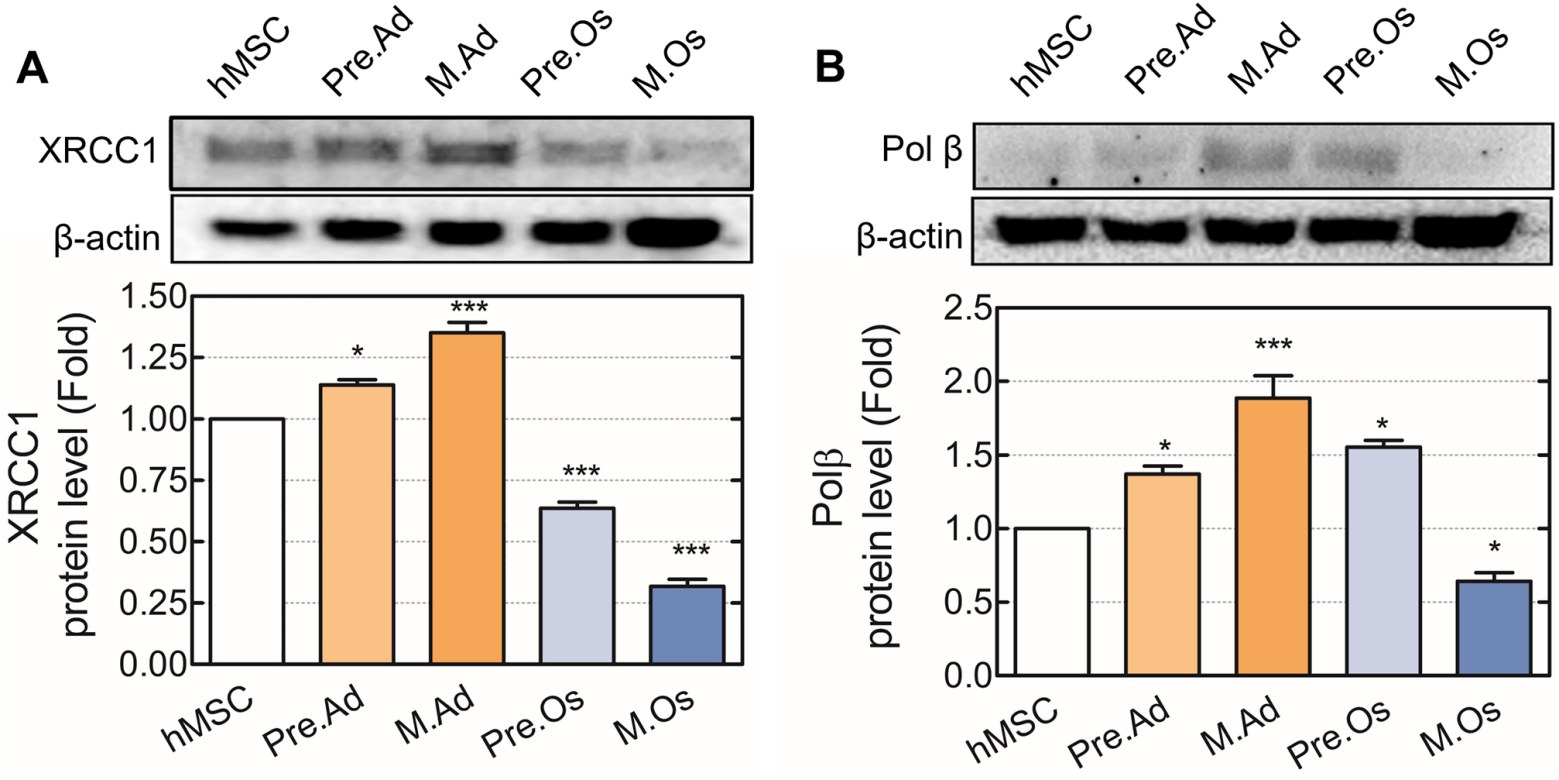
Expression of XRCC1 and Polβ during the adipogenic and osteogenic differentiation of hMSCs. The protein levels of (**A**) XRCC1 and (**B**) Polβ were evaluated using cell extracts from hMSCs, pre-adipocytes, mature adipocytes, pre-osteoblasts, and mature osteoblasts. Full-length blots/gels are presented in Supplementary Fig. 3. *p < 0.05, **p < 0.01, compared with the MSC group. hMSC, proliferating hMSC; Pre.Ad, pre-adipocytes; M.Ad, mature adipocytes; Pre.O, pre-osteoblast; M.Os, mature osteoblast.

AP sites, which result in the incision of damaged bases by a mono-functional glycosylase (e.g., APNG or UDG), are cleaved by APE1 and generated to the 5′-dRP during a base excision repair pathway^43^. After the reaction of APE1, repair proteins are stabilized by scaffold proteins such as XRCC1^44^. XRCC1 interacts with LIG3, PARP1, and DNA polymerase β (polβ) that coordinately perform repair and ligation of DNA^45^. XRCC1, not known for its enzymatic activity, is relevant to the repair efficiency of strand breaks because it participates in enzyme interactions. Especially in alkyl-damaged DNA correction of the BER pathway, XRCC1 and polβ have been reported to be responsible for protection against DNA strand breaks induced by MMS^46^. The protein expression of XRCC1 was increased two-fold in adipogenesis, while it was decreased in osteogenesis. Similarly, Polβ was also increased two-fold in adipogenesis and decreased in mature osteoblasts (Fig. 4). These results indicate that repair intermediates regulate the BER pathway and accumulate more DSBs in hMSCs than in adipocytes.

**Figure 5:**
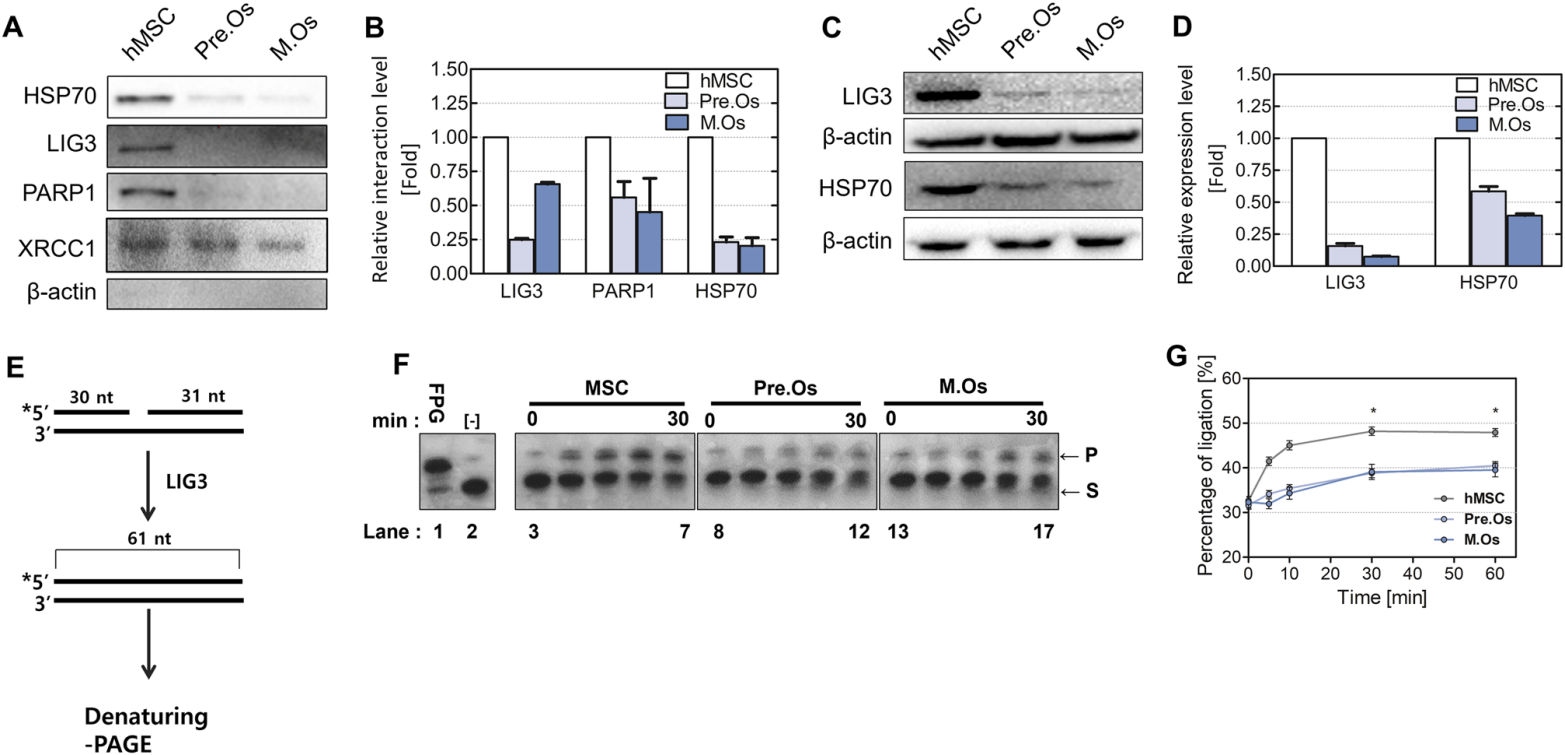
XRCC recruitment rate and ligation activity during the osteogenesis of hMSCs. Hsp70, LIG3, and PARP1 interact with the scaffold protein XRCC1. (**A**) The protein–protein interaction among Hsp70, LIG3, PARP1, and XRCC was evaluated via co-immunoprecipitation. Full-length blots/gels are presented in Supplementary Fig. 4. (**B**) Relative interaction levels with XRCC1 are quantified and plotted into a graph. (**C**) LIG3 and HSP70 levels were analyzed using cell extracts from hMSCs or differentiated cells and (D) plotted into a graph. Full-length blots/gels are presented in Supplementary Fig. 5. (**E**) Schematic representation of the ligation activity assay. (**F**) LIG3-activity assay was performed using cell extracts from hMSCs, p. Os, and m. Os. The relevant time points are indicated above each sample. Each blot was electrophoresed using mini-gels. (**G**) Each activity was quantified and plotted into a graph. T4 Lig is the positive control that contains T4 ligase from *Enterobacteria phage T4,* and [-] represents the negative control. hMSC, proliferating hMSC; Pre.O, pre-osteoblast; M.Os, mature osteoblast S, Substrate; P, Product. *Represents significant differences as compared with the MSC group.

To evaluate the correlation between XRCC1 and repair protein complex in undifferentiated hMSC differentiated osteoblast, the binding affinity of proteins that compose the complex XRCC1 was observed. The interaction between XRCC1 and repair proteins decreased during the osteogenic differentiation of hMSC (Fig. 5A and B). In differentiated osteoblasts, reduced protein interaction between PARP1 and XRCC1 was observed. The interaction between LIG3 and XRCC1 declined along the same line as PARP1. These reduced interactions lead to instability of LIG3 in osteoblast, resulting in attenuated DNA repair activity and decreased cell viability.

As shown in Fig. 5, the LIG3 expression level dramatically decreased during the osteogenic differentiation, and there was no significant difference in the expression level of LIG3 between pre-osteoblast and mature osteoblast (Fig. 5C and D). To evaluate the activity of LIG3, an enzymatic ligation assay was performed using whole cell extracts from each phase of differentiation and DNA substrate labeled by γ-^32^P-ATP containing nick lesion. To determine the initial rates of LIG3 activity during differentiation, time-course enzymatic activity reactions were conducted for various incubation times. Cell-free extracts from hMSC and each phase of differentiated cells showed significantly different ligation activity. The activity of LIG3 decreased during the osteogenic differentiation. In summary, the expression and activity level of LIG3 significantly reduced during the osteogenic differentiation of hMSC. In pre-osteoblast, declined LIG3 activity might cause cell death during recovery time when the cells are exposed to MMS. Additionally, the high activity of LIG3 in hMSCs might protect them from cell death during the recovery time. These results indicate that the expression and activity level of LIG3 directly influences the cell viability during the repair of DNA damage caused by an alkylating agent, and low activity level of LIG3 can generate an accumulation of unrepaired DNA strand break.

## Discussion

Bone marrow-derived hMSCs have the potential to differentiate into various cell types, such as adipocytes, osteoblasts, and neuronal cells^47^. This study aimed to determine how DNA damage response stimulated by alkylating agent, MMS, is regulated during adipogenic differentiation in hMSCs. We showed that osteoblasts were more susceptible than other cells, and hMSCs were more susceptible than adipocytes to the MMS-induced damage. To determine the effect of BER pathway modulation on MMS-induced DNA damage repair during adipogenesis and osteogenesis, we analyzed the expression levels of BER-processing enzymes, such as UDG and XRCC1, and the activities of these enzymes via cleavage assay. Additionally, we found that osteoblasts are the most vulnerable cells to MMS-induced DNA damage, whereas adipocytes are quite resistant. Bone diseases, including osteopenia and osteoporosis, are considerably recognized in cancer patients treated with alkylating chemotherapeutics^17^, whereas the repair mechanisms of bone cells damaged by alkylating agents are poorly understood. So, investigating the modulation of repair mechanisms during the osteogenic differentiation of hMSC is critical to using better chemotherapeutic agents. Our results showed that osteoblasts are most sensitive to the MMS, and undifferentiated hMSCs are more sensitive to the alkylating agent MMS, compared with adipogenic differentiated cells, as suggested by the accumulation of DNA strand breaks. One possible explanation for these results is that the repair intermediates, such as DNA strand breaks, are accumulated as DNA damage repair proteins are downregulated during osteogenic differentiation. Cecilia et al.^48^ discovered that DNA single-strand break intermediate formation occurred in 2 h after DNA damage agents were eliminated from the medium in PARP knockdown cells. This study suggests that the expression or activity level of DNA repair proteins affects cell viability by modulating the DNA repair process during the recovery time.

APE1 is responsible for cleaving the DNA backbone and can generate DNA strand breaks. The levels of XRCC1, polβ, and UDG were increased in adipocytes but not in hMSCs. The activity of UDG was increased during the adipogenesis of hMSCs (Fig. 3C and D). UDG deficiency could spontaneously lead to mutations in mammalian cells because uracil causes various mutation reactions in cells. In addition, human UDG has been shown to have a crucial function in the excision of DNA base lesions, including alloxan, 5-OH-uracil, and isodiauric acid, produced by free radicals. Interestingly, the protein level and enzymatic activity of the bifunctional glycosylase OGG1 and endonuclease APE1 did not significantly differ (Sup. Figs. 1 and 2), whereas those of UDG were significantly regulated during the differentiation (Fig. 3C–G). Previous Studies have shown that UDG activity and APE1 activity react differently depending on the type of DNA damage. UDG activity increased for oligos containing RcdG, while hAPE1 activity increased for oligos containing ScdA and ScdG^49^. Our results indicate that UDG has an important role than any other glycosylase in the repair pathway of MMS-mediated DNA damage.

Pol β was upregulated during adipogenic differentiation and downregulated during osteogenesis (Fig. 4). Deficiency of polβ results in hypersensitivity to alkyl damage and chromosomal abnormality. However, non-proliferative cells, which are deficient in polymerase, are tolerant to DNA damage, while polymerase-deficient cells in the G1 phase preparing for DNA replication are sensitive^50^. In addition, polβ protein level differs among cell lines, proliferating, and differentiated cells^51^. Thus, the regulation of polβ protein levels in differentiated cells might be a result of alterations in the intercellular microenvironment following differentiation. In addition, XRCC1 is known to have diverse regulations of the ligation process. Previously, XRCC1 has been shown to stabilize ligase III and promote the activity of polβ in an ATP-abundant environment^52^. The intercellular environment of adipocytes is known to be ATP-abundant^53,54^. To summarize these reports, the abundance of XRCC1, as well as ATP in the intercellular environment of adipocytes, can facilitate the activity of polβ and LIG3 during the adipogenesis of hMSCs. hMSCs regulate DDR protein abundance to be susceptible to DNA mutation because hMSCs are highly responsible for maintaining genome homeostasis^55^. In the bone turnover process, osteoblast apoptosis occurs, which is pertinent to skeletal development and makes balance with osteogenic differentiation to maintain skeletal structure^56^, whereas adipose tissue has a slow turnover rate^57^. In other words, it is supposed that it is less important for osteoblast to maintain their genomic integrity than hMSCs, so they express low levels of polβ and XRCC because non-dividing cells are tolerant to DNA damage through G0 phase arrest^58,59^.

Here, we proved that DNA repair proteins are regulated during the adipogenic and osteogenic differentiation of hMSCs. A decrease in XRCC1 level affects the activity of BER proteins because XRCC1 is a scaffold protein that recruits BER proteins^60^. It is reported that XRCC1, DNA ligase III, flap endonuclease 1 (FEN1), and poly(ADP-ribose) polymerase are important proteins for both BER and the repair of alkylating agent induced lesions^61^. Eva et al.^52^ have reported that a decrease in the protein levels of LIG3 was observed in the cells in which XRCC1 expression was inhibited by transfection of small interfering RNA (siRNA). XRCC1 and LIG3 interact to form a stable complex, and LIG3 is regulated via poly-ubiquitylation as XRCC1 is degraded by the E3 ubiquitin ligase CHIP^45^. We confirmed that the protein and activity levels of LIG3 declined during the osteogenic differentiation (Fig. 5E–G). Additionally, an interaction between XRCC1 and LIG3 was impaired in differentiated osteoblasts (Fig. 5A and B). The instability of XRCC1 and a decrease in the interaction between LIG3 and XRCC1 can lead to the degradation and low activity of LIG3. Our results show that HSP70 and PARP1 have a decreased interaction with XRCC1. HSP70 transport the proteins into the nucleus to repair DNA damage or protect DNA from further damage^62^. In addition, PARP1 acts as a DNA damage sensor. Our results indicate that the DNA damage sensing and response by HSP70 and PARP1 are decreased during osteogenesis.

DNA repair proteins are downregulated during the differentiation of mitotic cells, such as hMSCs, into post-mitotic cells, including muscle and neuronal cells^26,28,63^. In the same manner, we covered that DNA repair was downregulated during the osteogenic differentiation of hMSCs. The expression level of XRCC1 significantly decreased in mature osteoblasts. The ligation step in differentiated osteoblast is downregulated by the low level of expression and activity of LIG3, which is caused by the decline of the interaction between XRCC1 and LIG3. These considerable changes in DNA repair proteins during the osteogenic differentiation of hMSC are causative of the transition of cell viability of osteoblast during recovery time. Judging from our research, alkylating agents used in chemotherapy can impair the viability of osteoblasts during the recovery phase, and thus damaged osteoblasts can cause the collapse of bone homeostasis and ultimately lead to bone diseases, including osteoporosis. During the osteogenic differentiation of hMSCs, these investigations into the repair pathway and its regulatory mechanisms in response to alkylating agents will provide the key lead for the proper use of anti-cancer drugs such as alkylating agents.

In this study, we suggest the correlation between DNA repair capacity and the sensitivity of differentiated cells to MMS-induced alkylation damage. The MMS-induced cellular damage at each phase of differentiation was tolerated in adipocytes, whereas osteoblasts were more sensitive to damage than hMSCs. The formation of DNA DSBs detected by γH2AX foci is also alleviated in adipocytes but slightly decreased in osteoblasts compared with hMSCs. The UDG activity and protein level were upregulated in adipocytes. APE1 and OGG1 expression levels and activity showed no significant changes in differentiated cells; however, the level and activity of UDG were significantly increased in adipocytes and decreased in osteoblasts. The level of XRCC1 and polβ, crucial to the repair and ligation step, gradually increased in adipogenesis, while those levels and recruitment declined during osteogenesis. This study determined the modulation of alkyl DNA damage response focused on BER pathway regulation during adipogenic and osteogenic differentiation. However, mechanisms of the overall BER pathway in hMSCs differentiation need to be investigated in more depth to clarify the effect on genome integrity and finally apply to stem cell therapy or cancer therapy.

## Materials and methods

### Cell culture

hMSCs were purchased from the American Type Culture Collection (ATCC, Manassas, VA, USA) and the characterization of MSC was performed by ATCC. Cell morphology, proliferation rate, and surface marker expression were all examined for characterization and described in the manufacturer’s certificate. All cells are negative for CD14, CD19, CD34, CD45 and positive for CD29, CD44, CD73, CD90, CD105 and CD166, which meet to International Society for Cellular Therapy(ISCT) guidelines. The cells were maintained in the stemMAC MSC expansion medium (Miltenyi Biotec, Germany) containing 100 units/ml penicillin and 100 μg/ml streptomycin (Gibco, NY, USA).Considering the cells received from ATCC as the first passage (passage 1), the cells used in the experiment were the passage 5. The passage 5 MSCs seeded on the plates and cultured for up to 21 days for adipogenesis and osteogenesis. Previous reports demonstrate that the passage 5 shows early passage properties, which maintains differentiation capacity and required MSC phenotypes for both positive and negative CD markers^64,65^. Confluent cells were detached using Accutase (Innovative Cell Technologies, CA, USA). For adipogenic differentiation, cells were seeded at a density of 2 × 10^4^ cells/cm^2^ on culture dishes. After cells reached 100% confluence, the culture medium was replaced with the adipogenic induction medium (AIM), composed of Dulbecco’s modified Eagle’s medium (DMEM)-high glucose (4.5 g/l, Gibco, NY, USA), 100 units/ml penicillin, 100 μg/ml streptomycin, and 10% defined fetal bovine serum (Gibco, NY, USA) i.g. plus 1 μM dexamethasone, 100 μM indomethacin, 500 μM isobutyl-methylxanthine (Sigma-Aldrich, MA, USA), and 10 μg/ml insulin (Welgene, Daegu, Republic of Korea). The cells were cultured at 37 ℃ in a humidified atmosphere containing 5% CO_2_ and 95% air. For the osteogenic differentiation of hMSC, cells were seeded at 2 × 10^4^ cells/cm^2^ and cultured in DMEM-low glucose (1 g/l, Gibco, NY, USA) with 10% fetal bovine serum and 1% penicillin–streptomycin, containing 1 μM Dexamethasone (Sigma-Aldrich, MA, USA), 10 mM β-Glycerophosphate (Sigma-Aldrich, MA, USA) and 50 μM l-Ascorbic acid-2-phosphate (Sigma-Aldrich, MA, USA). All media was changed twice per week.

### Oil-Red-O staining

Adipogenic differentiation was evaluated via Oil-Red-O (ORO) staining (Sigma-Aldrich, MA, USA). Cells were washed twice with PBS and then fixed with 10% formalin (Sigma-Aldrich, MA, USA) for 10 min. After fixation, ORO staining solution was applied at room temperature (RT) for 30 min to stain the lipid vesicles. To perform the quantification of the remaining ORO, the dye was eluted using isopropanol (Sigma-Aldrich, MA, USA) containing 4% igepal (Sigma-Aldrich, MA, USA), and the absorbance was measured at 492 nm^14^.

### Alizarin-Red-S staining

Osteogenic differentiation was evaluated using Alizarin Red S (ARS) staining. The culture medium was removed and washed twice with calcium-free 1× PBS. For cell fixation, 3.7% formaldehyde solution (Sigma-Aldrich, MA, USA) was treated for 15 min at RT. The fixed cells were stained with 2% ARS (Sigma-Aldrich, MA, USA) solution for 45 min at RT in the dark. The stained cells were stored in 1× PBS and observed under a light microscope. Furthermore, to calculate the intensity of the calcium nodules, 10% cetylpiridinium chloride (CPC) (Sigma-Aldrich, MA, USA) solution was used as the elution buffer. The absorbance of CPC solution was measured at 570 nm^66^.

### Reverse transcription-quantitative polymerase chain reaction (RT-qPCR) analysis

Total RNA was extracted by Trizol reagent (Life Technologies, CA, USA) according to the manufacturer’s instructions and then quantified using a Nanovue spectrophotometer (GE Healthcare, IL, USA). cDNA was synthesized from 2 μg of total RNA with an M-MLV (Moloney Murine Leukemia Virus) Reverse Transcriptase (ELPIS-BIOTECH, Daejeon, Republic of Korea) according to the manufacturer’s instructions and used in RT-qPCR (CFX Connect™ Real-Time PCR Detection System; Bio-Rad, CA, USA) for analysis of target genes. RT-qPCR was performed using SYBR Green PCR Master Mix (KAPA, MA, USA) according to the manufacturer’s protocol. The reaction conditions were 95 ℃ for 3 min, then cycling for 40 cycles of 95 ℃ for 10 s, 60 ℃ for 10 s, and 72 ℃ for 10 s^67^.

### Cell-viability assay

Cell viability was evaluated using the MTT (3-[4,5-dimethylthiazol-2-yl]-2,5-diphenyltetrazolium bromide) method. Each phase of differentiated cells and proliferating MSCs treated with various concentrations of MMS for 30 min were washed with PBS and then incubated with DMEM for 1 h or 24 h. Then, the cells were incubated with the medium containing 5 mg/ml MTT for 3 h at 37 ℃. After the medium was discarded, dimethyl sulfoxide was added to elute formazan. After 10 min of incubation, the absorbance was measured using a microplate spectrophotometer (TECAN, Switzerland) at a wavelength of 570 nm^68^.

### Immunofluorescence analysis

Cells were fixed in 10% formamide for 15 min at room temperature after each day of differentiation. Then, their membranes were permeabilized by PBS containing 0.25% Triton X-100 (Sigma-Aldrich, MA, USA) for 10 min. Cells were blocked with Tris-buffered saline-tween 20 (TBST) containing 1% bovine serum albumin (BSA; Sigma-Aldrich, MA, USA) for 30 min, primary antibodies (Millipore, MA, USA) for 1 h and Alexa 488-conjugated secondary antibody (Cell Signaling Technology, MA, USA) for 1 h in the dark. Then, 4,6-diamidino-2-phenylindole (DAPI; Sigma-Aldrich, MA, USA) was applied, and fluorescence images were obtained via confocal microscopy (Nikon, Japan)^68^.

### Enzyme extraction to assess BER-pathway activity

The cell extracts for enzymatic activity assay were resuspended in 20 mM HEPES–KOH (pH 7.6), 100 mM NaCl, 1 mM dithiothreitol (DTT), 0.1 mM ethylenediaminetetraacetic acid (EDTA), 1 mM phenylmethylsulfonyl fluoride, 10% (v/v) glycerol, and protease inhibitor cocktail (Sigma-Aldrich, MA, USA). The extracted protein for in vitro enzyme assay was obtained by centrifugation at 16,200 rpm at 4 ℃. The aliquots of the soluble protein were stored at – 70 ℃ until use. The extracted protein concentration was determined using a Bio-Rad Protein Assay reagent (Bio-Rad, CA, USA). Bovine serum albumin (Amersham Biosciences, UK) was used as the standard.

### 5′-end–labeling

Oligonucleotides were synthesized and then purified through a high-pressure liquid chromatograph from Operon Technologies. For the APE1 activity assay, a 30-mer DNA substrate containing THF at position 14 was labeled on the 5*′*-end by T4 polynucleotide kinase using [γ-^32^P] ATP at 37 ℃ for 1 h. The ^32^P-labeled THF-containing DNA substrates were passed through a Micro Bio-Spin 30 chromatography column (Bio-Rad, CA, USA) following the manufacturer’s protocol. Then, the ^32^P-labeled DNA substrates were annealed to the complementary DNA substrates in Tris–EDTA (TE) buffer by incubation at 80 ℃ for 3 min, followed by slow cooling to room temperature. For the glycosylase activity assay, 30-mer DNA substrates containing a uracil residue at position 14 were labeled using the same procedure mentioned above.

### Assessment of DNA-glycosylase activity

UDG activity was determined in a reaction mixture (10 µl) that contained 25 mM Hepes–KOH (pH 8.0), 50 mM KCl, 1 mM DTT, 0.5 mM EDTA, 0.1 mg/ml BSA, and 1 nM 5*′*-end labeled duplex DNA substrates containing a uracil residue. The reactions were initiated by adding 50 ng cell extract and then incubated at 37 ℃. Aliquots of each reaction were withdrawn at the 0, 3, 5, 10, 20, and 30 min. The reaction was terminated by transferring the reaction mixture to 0 ℃. The apurinic/apyrimidinic (AP) site generated by either enzymatic uracil removal from the DNA substrates was hydrolyzed by adjusting the mixture to 100 mM NaOH and then incubating at 70 ℃ for 10 min. The reactions were terminated by adding the formamide-loading buffer (95% formamide, 20 mM EDTA, 0.01% bromophenol blue, and 0.01% xylene cyanol). The DNA products were resolved using electrophoresis in a 15% denaturing polyacrylamide gel containing 7 M urea in 90 mM Tris, 90 mM boric acid, and 2 mM EDTA. The gels were dried using a gel dryer (Bio-Rad, CA, USA), and the product was visualized using autoradiography and quantified using the ImageQuant software v5.2 (Molecular Dynamics, CA, USA). The percentage of cleaved uracil residues was calculated from the number of products divided by the sum of total products and substrates.

### Ligation assay

A reaction mixture containing 10 nM oligonucleotide substrate in the ligation buffer (5 mM MgCl_2_, 40 mM Hepes–KOH (pH7.8), 0.5 mM DTT, 2 mM ATP, 0.36 mg/ml of BSA) and 1 μg of cell lysate was incubated at 37 ℃ for various periods. The reaction was terminated by the addition of formamide loading buffer (95% formamide, 20 mM EDTA, 0.01% bromophenol blue, 0.01% xylene cyanole) followed by incubation at 95 ℃ for 5 min. T4 ligase (New England Biolabs, UK) was used as a positive control, and no protein was added in the negative control. The DNA products were resolved using electrophoresis in a 15% denaturing polyacrylamide gel containing 7 M urea in TBE buffer (9 mM Tris, 9 mM boric acid, 0.2 mM EDTA).Gels were dried using a gel dryer (Bio-Rad, CA, USA), and the product was visualized using autoradiography and quantitated using the ImageQuant software v5.2 (Molecular Dynamics, CA, USA).

### Co-immunoprecipitation

The cells were lysed in 25 m M Tris–Cl (pH 7.0), 150 mM NaCl, 1% igepal CA-630, 1 mM EDTA, 5% glycerol, and 1× protease inhibitor cocktail (Sigma-Aldrich, MA, USA). The lysates were cleared off any debris via centrifugation (26,000 × g for 15 min at 4 ℃). A reaction mixture containing 1 μg of cell lysate and 0.2 μg primary antibodies was incubated overnight at 4 ℃. 40 μg protein G sepharose 4 fast flow (GE Healthcare, UK) was added to the reaction mixture, and the resulting slurry was incubated at 4 ℃ for 4 h. The sepharose beads were separated via centrifugation (400 × g for 5 min at 4 ℃), and the supernatants were removed. The SDS buffer was added to elute the protein complex from the sepharose beads, and the mixture was incubated at 95 ℃ for 5 min. The beads were separated via centrifugation (400 × g for 5 min at 4 ℃), and the supernatants were subjected to 8% SDS-PAGE for western blotting analysis^69^.

### Western blotting analysis

Cell lysates were prepared in RIPA buffer containing a protease inhibitor cocktail and phosphatase inhibitor cocktails 2 and 3 (Sigma-Aldrich, MA, USA). Total protein (20–50 µg) was resolved via 8% SDS-PAGE and subsequently transferred onto polyvinylidene difluoride membranes (GE Healthcare, IL, USA), which were later incubated with primary and secondary antibodies. The immunoreacting protein bands were visualized using an ECL western blotting detection reagent (GE Healthcare, IL, USA). Quantitative analysis of band intensities was performed using the Chemidoc Image Lab software (Bio-Rad, CA, USA)^67^.

### Statistical analysis

All the measurements were performed in triplicate, and all the values are expressed as means ± SEM. The results were subjected to an analysis of variance (ANOVA) by Tukey’s test to assess the statistical significance of differences. In this study, *p* < 0.05 was considered to indicate statistical significance.

### Supplementary Information

Supplementary Figures.

## Data Availability

All data generated or analyzed during this study are included in this published article.

## Abbreviations

AIM: Adipogenic induction medium
ALP: Alkaline phosphatase
APE1: AP endonuclease
APNG: *N*-Methylpurine-DNA glycosylase
ARS: Alizarin Red S
BER: Base excision repair ()
C/EBPβ: CCAAT/enhancer-binding protein-beta
DDR: DNA damage response
DSB: Double-strand break
FABP4: Fatty-acid binding protein
hBMMSCs: Human bone marrow-derived mesenchymal stem cells
MMS: Methanesulfonate
NEIL1: Nei-like DNA glycosylase 1
OCN: Osteocalcin
OGG1: 8-Oxo guanine glycosylase
ORO: Oil-Red-O
PARP1: Poly(ADP-ribose) polymerase-1
polβ: DNA polymerase β
PPARγ: Peroxisome proliferator-activated receptor gamma
Pref-1: Preadipocyte factor-1
Runx2: Runt-related transcription factor 2
UDG: Uracil-DNA glycosylase
XRCC1: X-ray repair cross-complementing protein 1
γH2AX: Histone H2AX
