# Supplementary material for: Regulation of base excision repair during adipogenesis and osteogenesis of bone marrow-derived mesenchymal stem cells
Source: Sci Rep. 2023 Sep 29;13:16384. doi: 10.1038/s41598-023-43737-z (PMC10542337; doi:10.1038/s41598-023-43737-z)
Supplement: Supplementary file 1 — Supplementary Figures. [file 41598_2023_43737_MOESM1_ESM.docx]

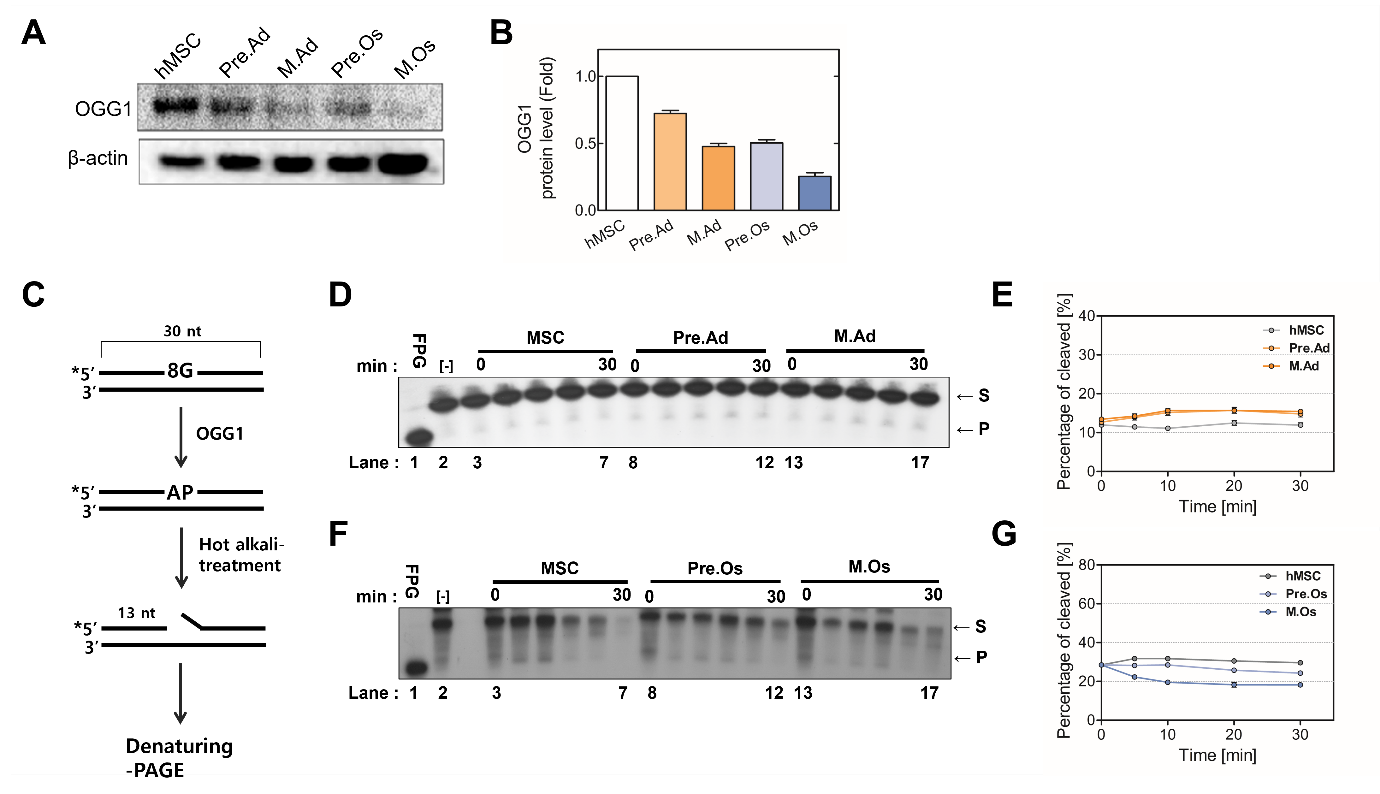


**Supplementary figure 1. Modulation of OGG1 expression and activity during the adipogenesis and osteogenesis of hMSCs.**

(A) Western blot showing the protein level of OGG1, and (B) quantitation results. (C) Schematic representation of the OGG1-activity assay. The 30-bp duplex DNA substrates containing single 8-oxo-guanine residue were labeled with [γ-32P]ATP at the 5'-end. The AP site generated through the enzymatic removal of the 8-oxo-guanine residue was hydrolyzed to produce the 13-bp cleaved products. Enzyme reactions contained 50 ng whole-cell extracts of (D) the adipogenic and (F) osteogenic differentiated cells at each phase in the reaction buffer. Purified *E. coli* FPG was used for generating 13-mer products as a positive control. The products were resolved using denaturing 15% PAGE and detected using autoradiography. (E and G) Each activity was quantified and plotted into a graph. MSC, proliferating hMSC; Pre.Ad, pre-adipocytes; M.Ad, mature adipocytes; Pre.O, pre-osteoblast; M.Os, mature osteoblast; S, substrate; P, product.


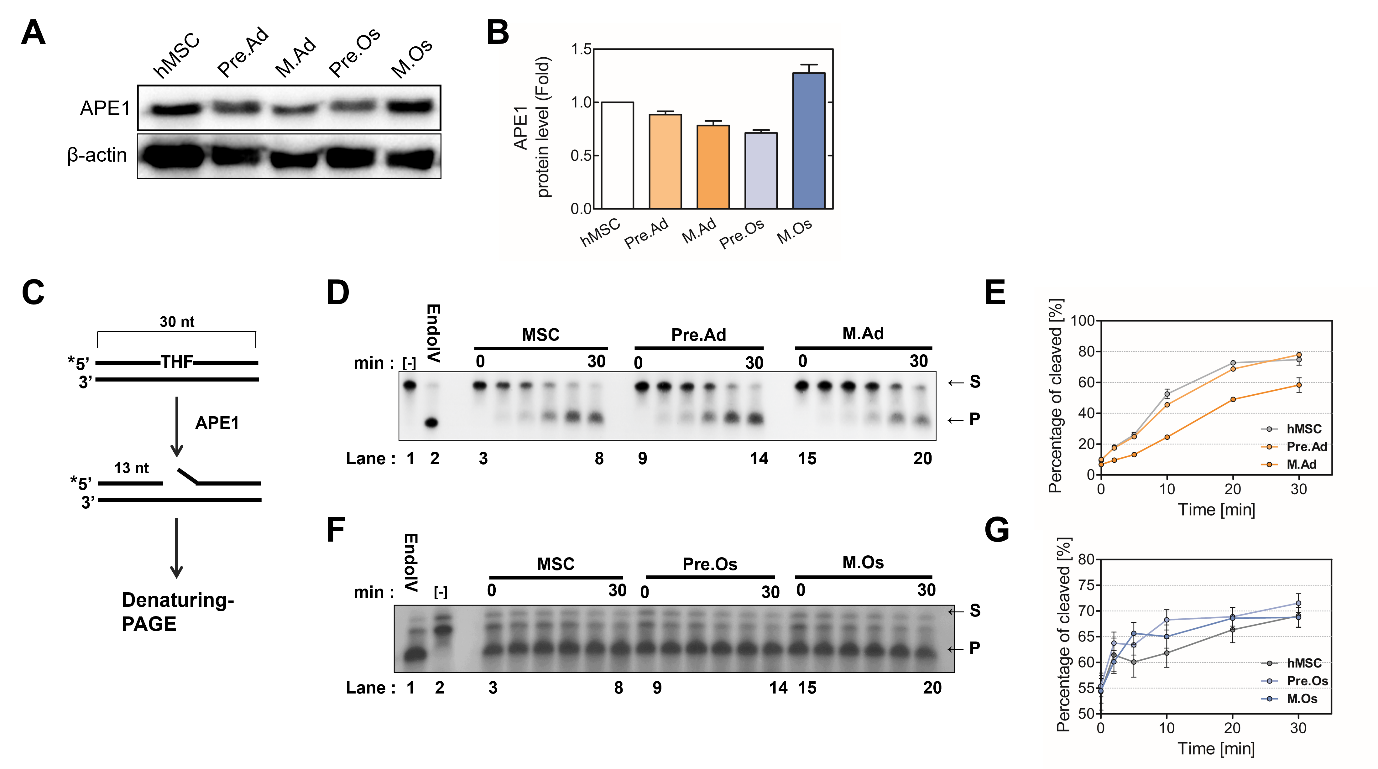


**Supplementary figure 2. Modulation of APE1 expression and activity during the adipogenesis and osteogenesis of hMSCs.**

(A) Western blot showing the protein level of APE1, and (B) quantitation results. (C) Schematic representation of the APE1-activity assay. The 30-bp duplex DNA substrates containing single THF residue were labeled with [γ-32P]ATP at the 5'-end. The AP site generated through the enzymatic removal of the uracil residue was hydrolyzed to produce the 13-bp cleaved products. Enzyme reactions contained 50 ng whole-cell extracts of (D) the adipogenic and (F) osteogenic differentiated cells at each phase in the reaction buffer. Purified *E. coli endonucleaseIV* (EndoIV) was used for generating 13-mer products as a positive control. The products were resolved using denaturing 15% PAGE and detected using autoradiography. (E and G) Each activity was quantified and plotted into a graph. MSC, proliferating hMSC; Pre.Ad, pre-adipocytes; M.Ad, mature adipocytes; Pre.O, pre-osteoblast; M.Os, mature osteoblast; S, substrate; P, product.


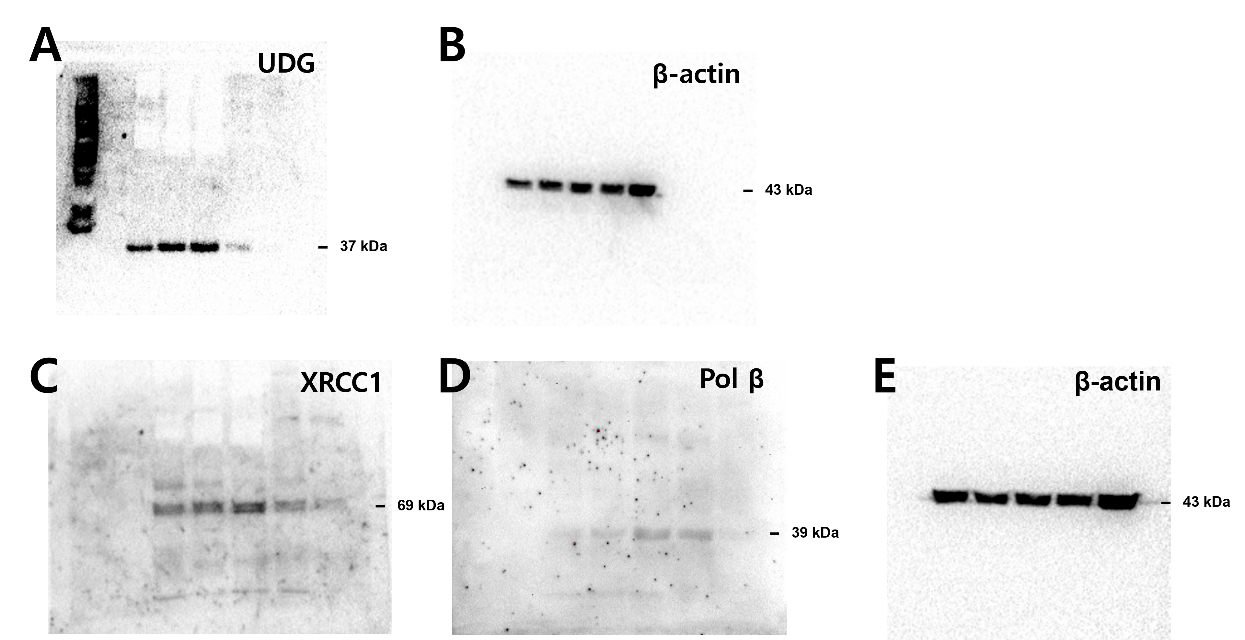


**Supplementary figure 3. Full-length blots/gels of figure 3 and 4**

(A) UDG in Fig 3A. (B) β-actin in Fig 3A and 4A. UDG and XRCC were detected using equal blot. (C) XRCC1 in Fig 4A. (D) Polβ and (E) β-actin in Fig 4B.


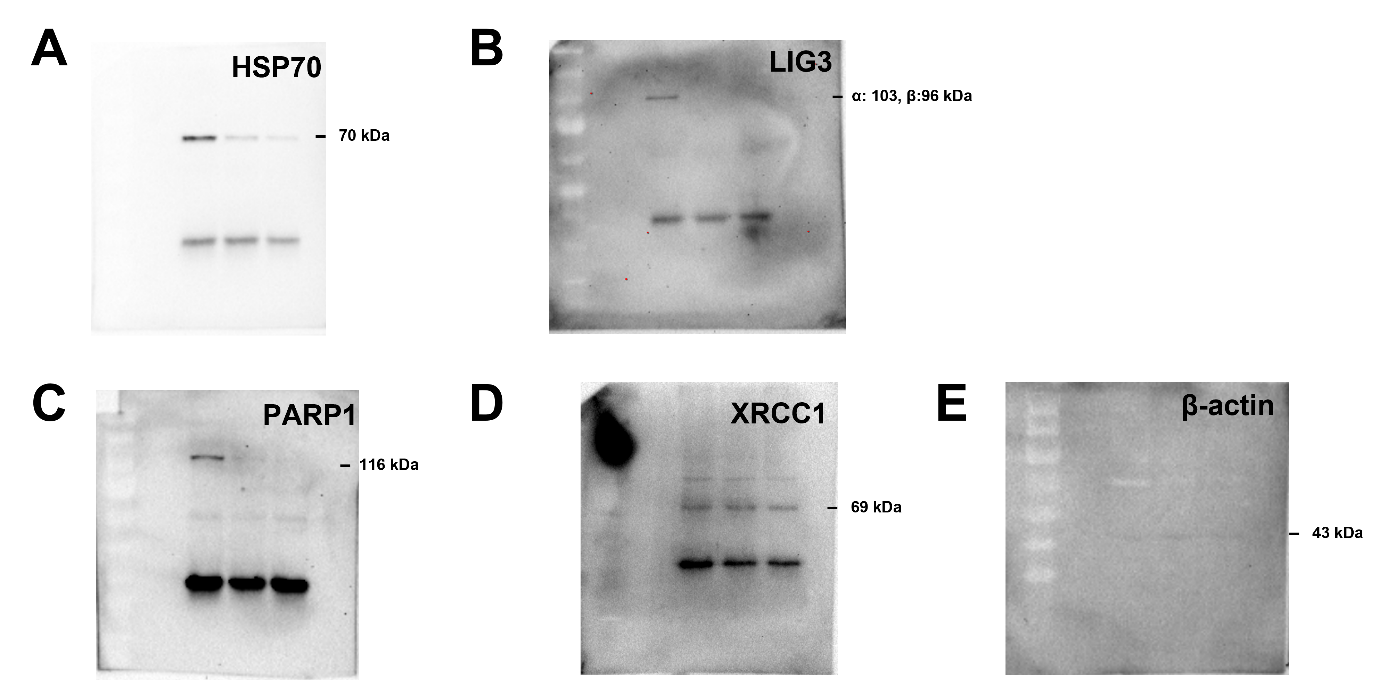


**Supplementary figure 4. Full-length blots/gels of figure 5A**

(A) HSP70, (B) LIG3, (C) PARP1, and (D) XRCC1 detected after immunoprecipitation and shown in Fig 5A. They are rabbit polyclonal antibodies. (E) β-actin in Fig 5A is mouse monoclonal antibody.


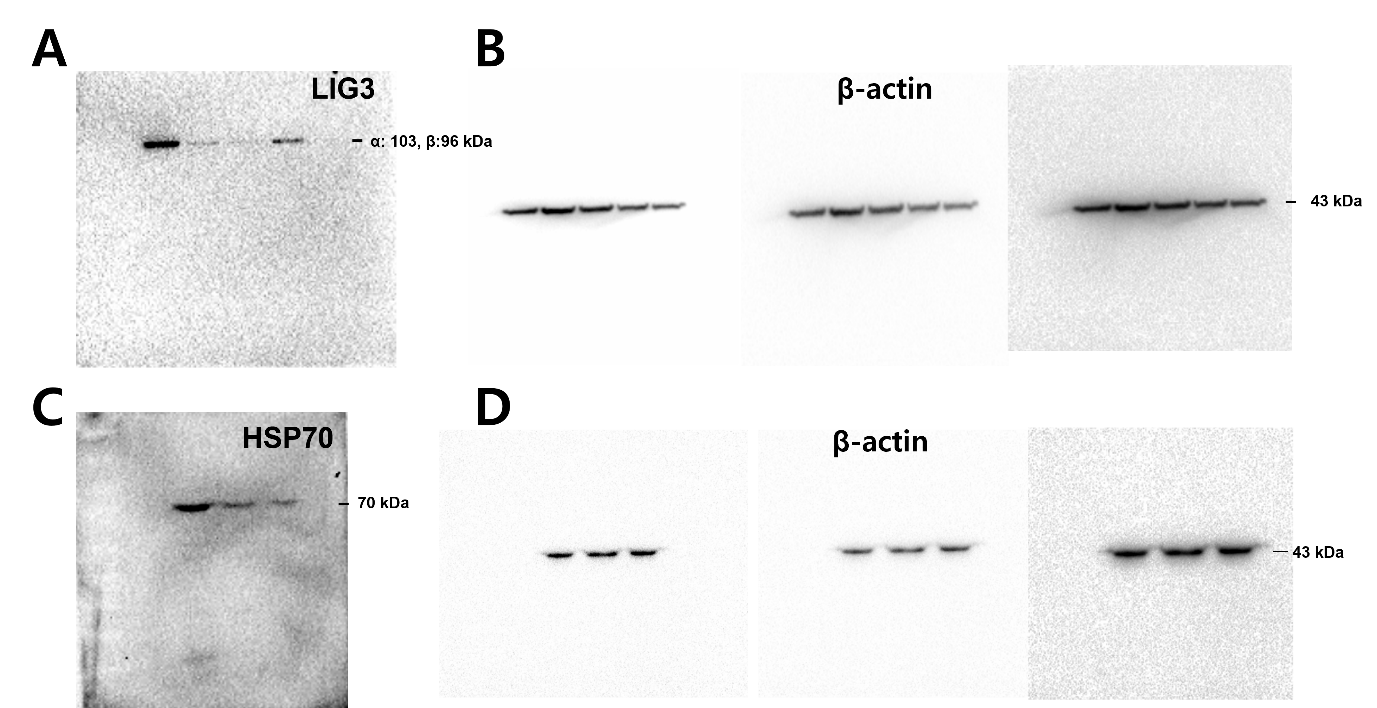


**Supplementary figure 5. Full-length blots/gels of figure 5B**

(A) LIG3 and (B) its β-actin in Fig 5B. (C) HSP70 and (D) its β-actin in Fig 5B. Multiple exposure images are shown in (B) and (D).


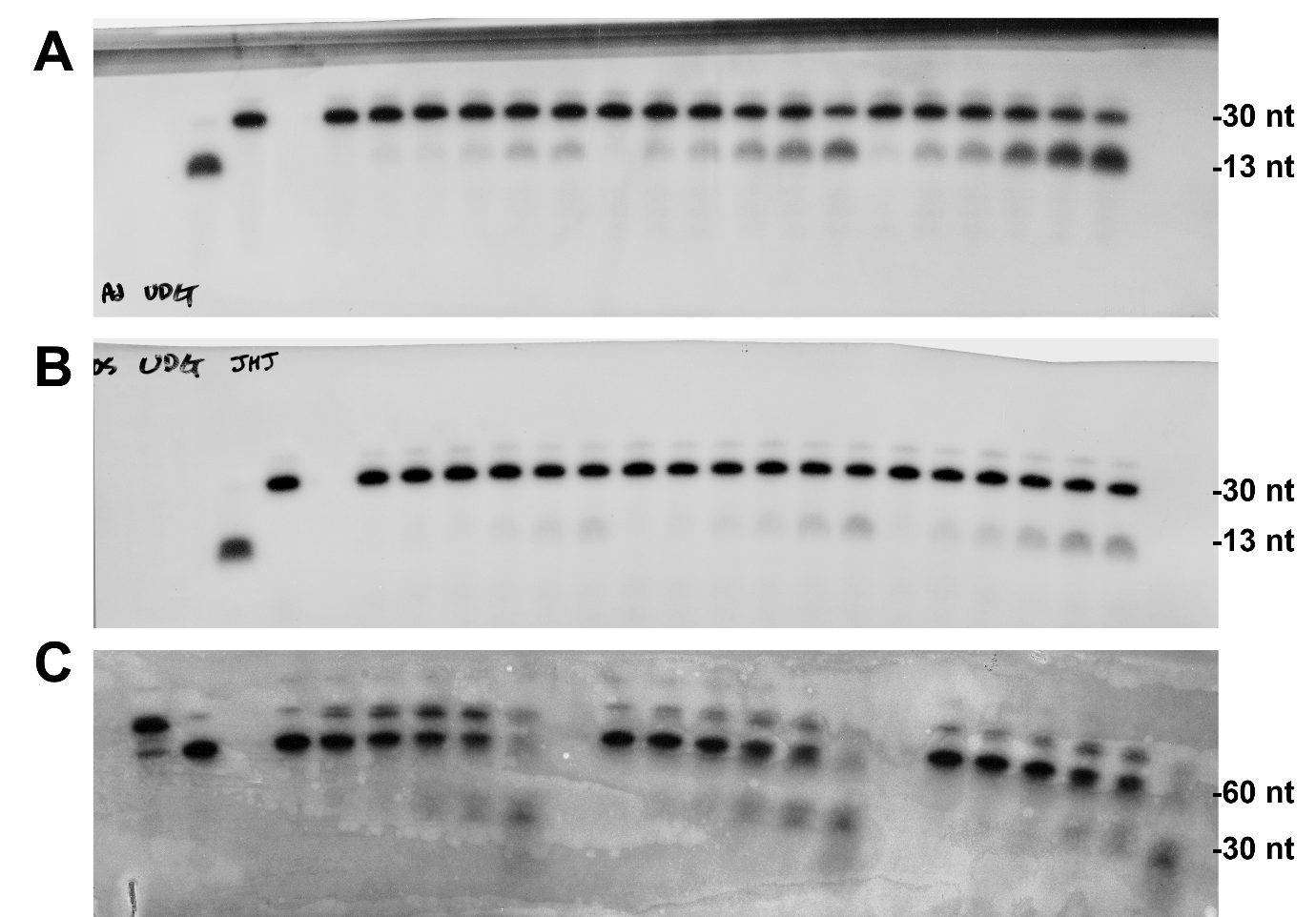


**Supplementary figure 6. Full-length blots/gels of figure 3D, 3F, and 5F**

The results of UDG activity of (A) adipocyte and (B) osteoblast are obtained by urea PAGE using 300 mm x 500 mm sequencing gel. (C) The results of the ligation assay are obtained by urea PAGE using 100 mm x 170 mm mini gels analyzed at the same time.


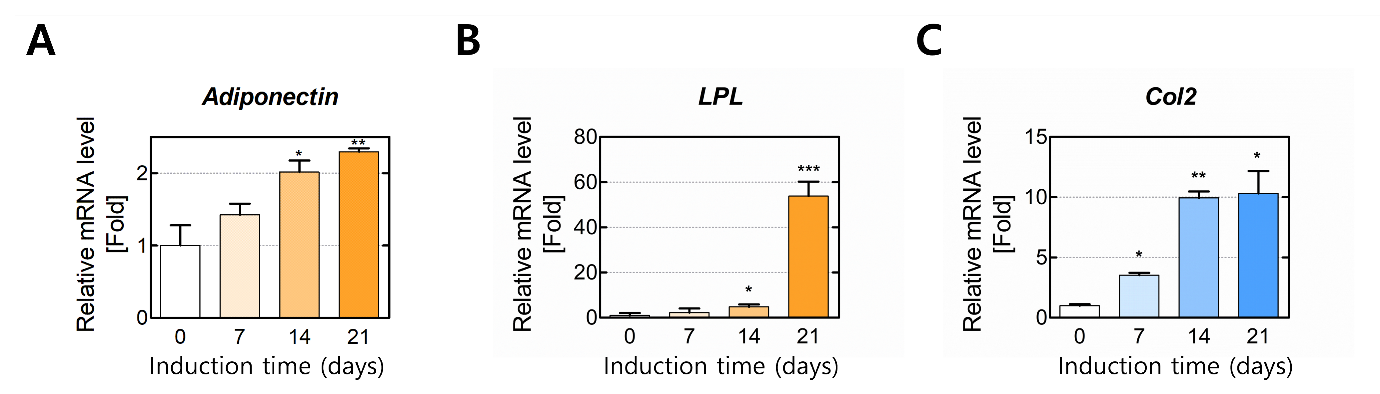


**Supplementary figure 7. Additional marker of mature adipocytes and osteoblasts**

Expression levels of (A) adiponectin and (B) LPL for adipocytes and (C) collagen2 for osteoblast markers. **p* < 0.05, ***p* < 0.01, and ****p* < 0.001 compared with the MSC group.


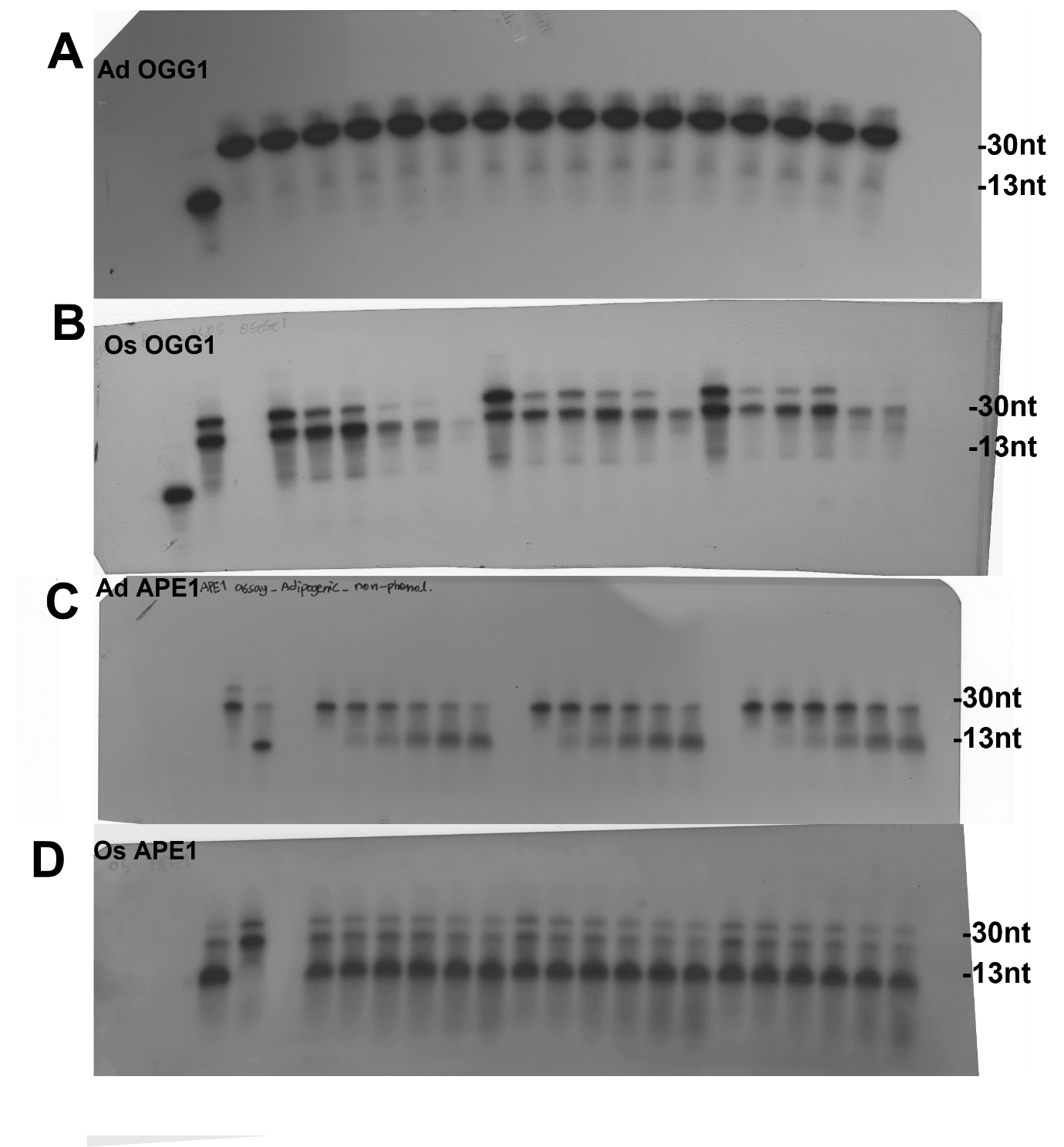


**Supplementary figure 8. Full-length blots/gels of supplementary figure 1D, 1F, 2D, and 2F**

The results of OGG1 activity of (A) adipocyte and (B) osteoblast are obtained by urea PAGE using 300 mm x 500 mm sequencing gel. The results of APE1 activity of (C) adipocyte and (D) osteoblast are obtained by urea PAGE using 300 mm x 500 mm sequencing gel.

**
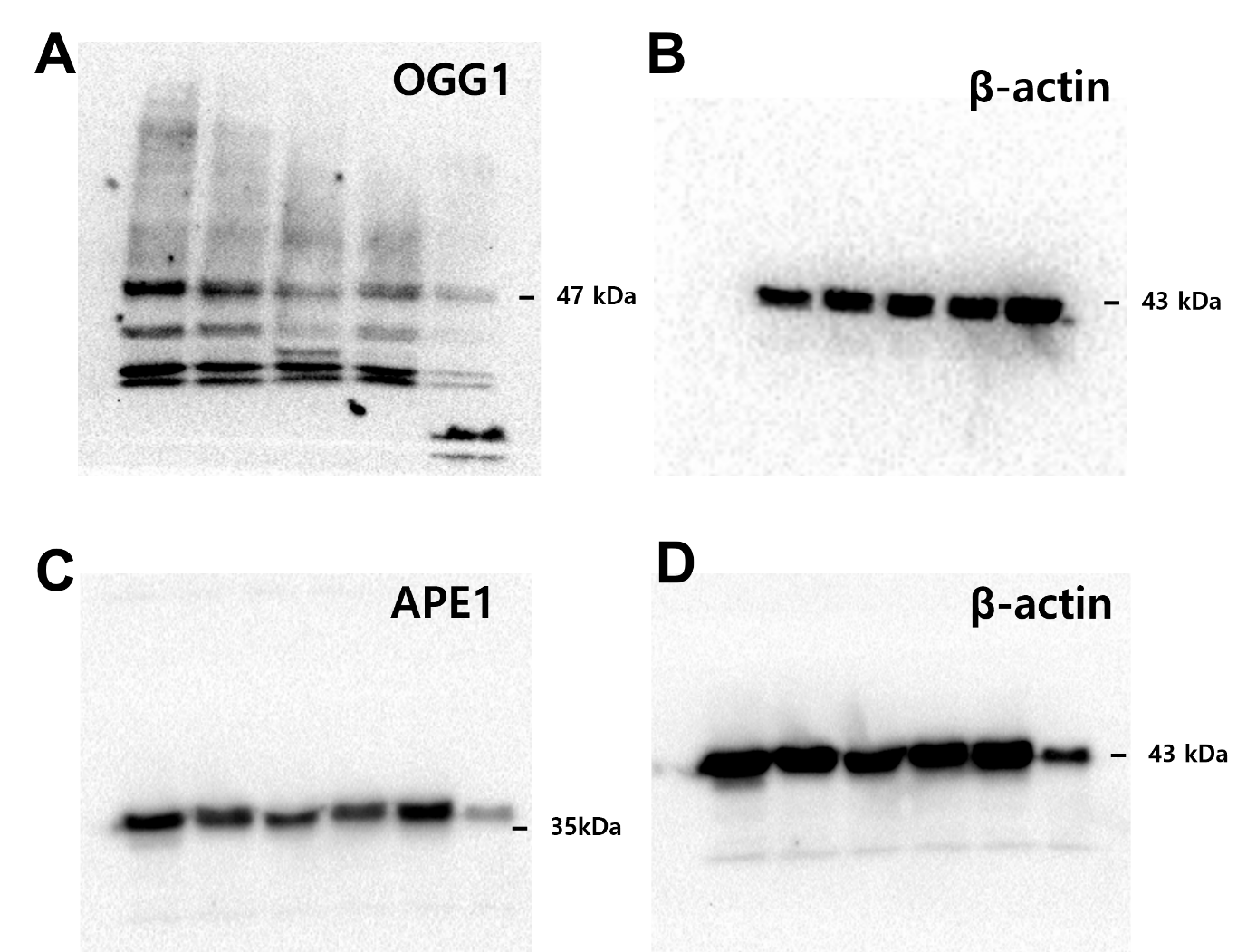
**

**Supplementary figure 9. Full-length blots/gels of supplementary figure 1A and 2A**

(A) OGG1 and (B) its β-actin in supplementary figure 1A. (C) APE1 and (D) its β-actin in supplementary figure 2A.


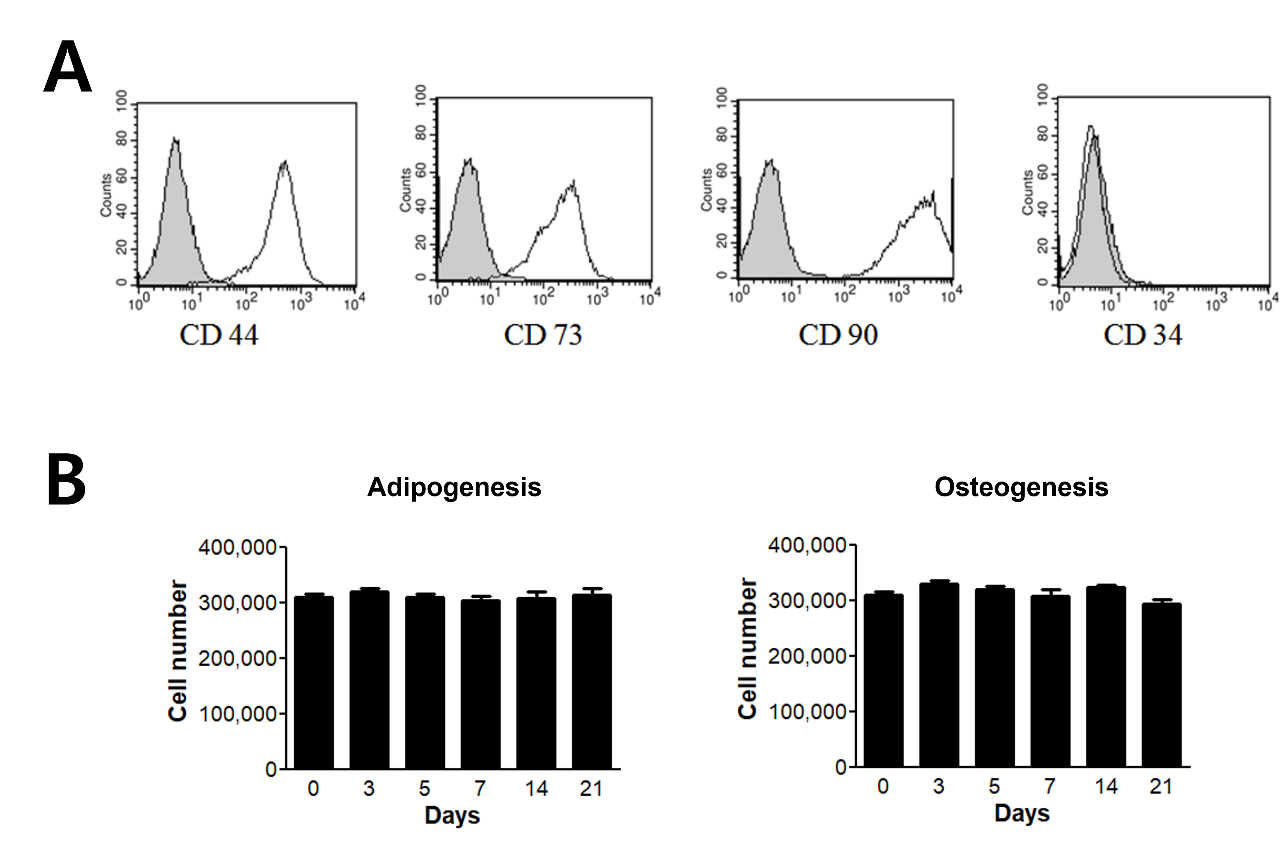


**Supplementary figure 10. MSC characterization and proliferation rate of MSC**

(A) The MSC markers were characterized before differentiation. (B) To validate the proliferation of MSC during differentiation, the cell numbers were assessed.
